# Household Secondary Attack Rates of SARS-CoV-2 Omicron Variant, South Korea, February 2022

**DOI:** 10.3201/eid2808.220384

**Published:** 2022-08

**Authors:** Do Sang Lim, Young June Choe, Young Man Kim, Sang Eun Lee, Eun Jung Jang, Jia Kim, Young-Joon Park

**Affiliations:** Korea Disease Control and Prevention Agency, Cheongju, South Korea (D.S. Lim, Y.M. Kim, S.E. Lee, E.J. Jang, J. Kim, Y.-J. Park);; Korea University Anam Hospital, Seoul, South Korea (Y.J. Choe)

**Keywords:** COVID-19, household, attack rate, South Korea, coronavirus disease, SARS-CoV-2, severe acute respiratory syndrome coronavirus 2, viruses, respiratory infections, zoonoses, vaccine-preventable diseases, booster

## Abstract

We studied the effect of booster vaccinations on reducing household transmission of SARS-CoV-2 B.1.1529 (Omicron) variant in a February 2022 sampling of contacts in South Korea. The secondary attack rate was lower for vaccinated versus unvaccinated contacts, and booster vaccination resulted in a lower incidence rate ratio.

Since its initial detection in November 2021, the SARS-CoV-2 B.1.1.529 (Omicron) variant has become the dominant strain in South Korea. Its emergence led to a large increase in the number of COVID-19 cases, mainly through household transmission (*1*,*2*). In this study, we sought to estimate the effect of booster vaccinations on reducing the household transmission of COVID-19 to guide current COVID-19 mitigation strategy.

This national, retrospective cohort study included all residents in South Korea with laboratory-confirmed SARS-CoV-2 infection reported during February 1–10, 2022. The background population was estimated as 53 million persons according to the 2021 census. Booster vaccinations with mRNA vaccines were provided in October 2021, reaching ≈30 million doses (60% of the total population) by February 2022. We retrieved epidemiologic data, merged with the national immunization registry of household contacts of persons infected with SARS-CoV-2, to describe the difference in secondary attack rates (SARs) by vaccination status. Details of the surveillance system, vaccination program, and dataset employed in this study are described in a previous study (*3*). Persons who had household contact with laboratory-confirmed SARS-CoV-2–positive patients underwent mandatory PCR testing, regardless of the presence of symptoms, and were put under active surveillance for 10 days. During the quarantine period, PCR testing was mandated when the household contact had symptoms, and testing was performed on day 9 or day 10 if the contact had no symptoms. 

We defined an index case-patient as a person with a positive SARS-CoV-2 test result determined through epidemiologic investigation who was most likely not infected in the household, a household contact as a person living in the same home as an index case-patient, and a household-infected case-patient as a person living in the same home as an index case-patient who had a positive PCR test result for SARS-CoV-2. We defined partly vaccinated persons as those who had received the first dose of a 2-dose vaccination regimen >14 days and fully vaccinated persons as those who had completed a 2-dose regimen of Pfizer-BioNTech (https://www.pfizer.com), AstraZeneca (https://www.astrazeneca.com), Moderna (https://www.moderna.com), or mix-and-match vaccines (time since vaccination >14 days) or those who completed a 1-dose regimen of the Janssen/Johnson & Johnson (https://www.janssen.com) vaccine (time since vaccination >28 days). We defined a booster dose as a third vaccination dose (>14 days since administration) after 2 doses of a primary vaccination series.

Data from the period February 1–10, 2022, revealed 163,581 household contacts of index case-patients with PCR-confirmed SARS-CoV-2 (Table). Within 10 days of active monitoring, 59,982 household contacts were confirmed to have SARS-CoV-2 infection, resulting in an SAR of 36.7%. Children 0–11 years of age had the highest SAR (55.1%), followed by adolescents 12–17 years of age (44%) and adults 30–39 years of age (44%) (p<0.001). The SAR was highest in contacts who were unvaccinated (53%), followed by those who received the Janssen vaccine (49%) or the AstraZeneca vaccine (37.2%). The SAR was comparatively lower in contacts who received the Pfizer-BioNTech vaccine (34.1%), the Moderna vaccine (32.7%), or a mix-and-match vaccine series (30.4%) (p<0.001). In examining the incidence rate ratio of household contacts according to the vaccination status of the SARS-CoV-2 index case-patients (Figure), we found that booster vaccination in household contacts resulted in a lower incidence rate ratio, irrespective of vaccination status of the index case-patient.

**Table:** Household contacts, household infected cases, and secondary attack rate of SARS-CoV-2 Omicron variant, South Korea, February 1–10, 2022

Characteristic	No. household contacts	No. household infection cases	Secondary attack rate, %
Total	163,581	59,982	36.7
Sex			
M*	80,145	27,595	34.4
F	83,436	32,387	38.8
Age group, y			
0–11*	18,456	10,173	55.1
12–17	13,266	5,839	44.0
18–29	26,243	8,497	32.4
30–39	15,920	7,006	44.0
40–49	31,477	12,497	39.7
50–59	33,920	9,302	27.4
60–74	18,037	5,056	28.0
>75	6,262	1,612	25.7
Vaccine type†			
Comirnaty/Pfizer-BioNTech*	87,296	29,808	34.1
Vaxzevria/AstraZeneca	1,638	610	37.2
Spikevax/Moderna	19,398	6,335	32.7
Jcovden/Janssen	261	128	49.0
Mix-and-match‡	26,780	8,144	30.4
Unvaccinated	28,208	14,957	53.0

*p<0.001. †Pfizer-BioNTech, https://www.pfizer.com; AstraZeneca, https://www.astrazeneca.com; Moderna, https://www.modernatx.com; Janssen/Johnson & Johnsoh, https://www.janssen.com. ‡Heterologous (mix-and-match) vaccinations with mRNA vaccines were provided to AstraZeneca-primed and Janssen-primed persons.

**Figure:**
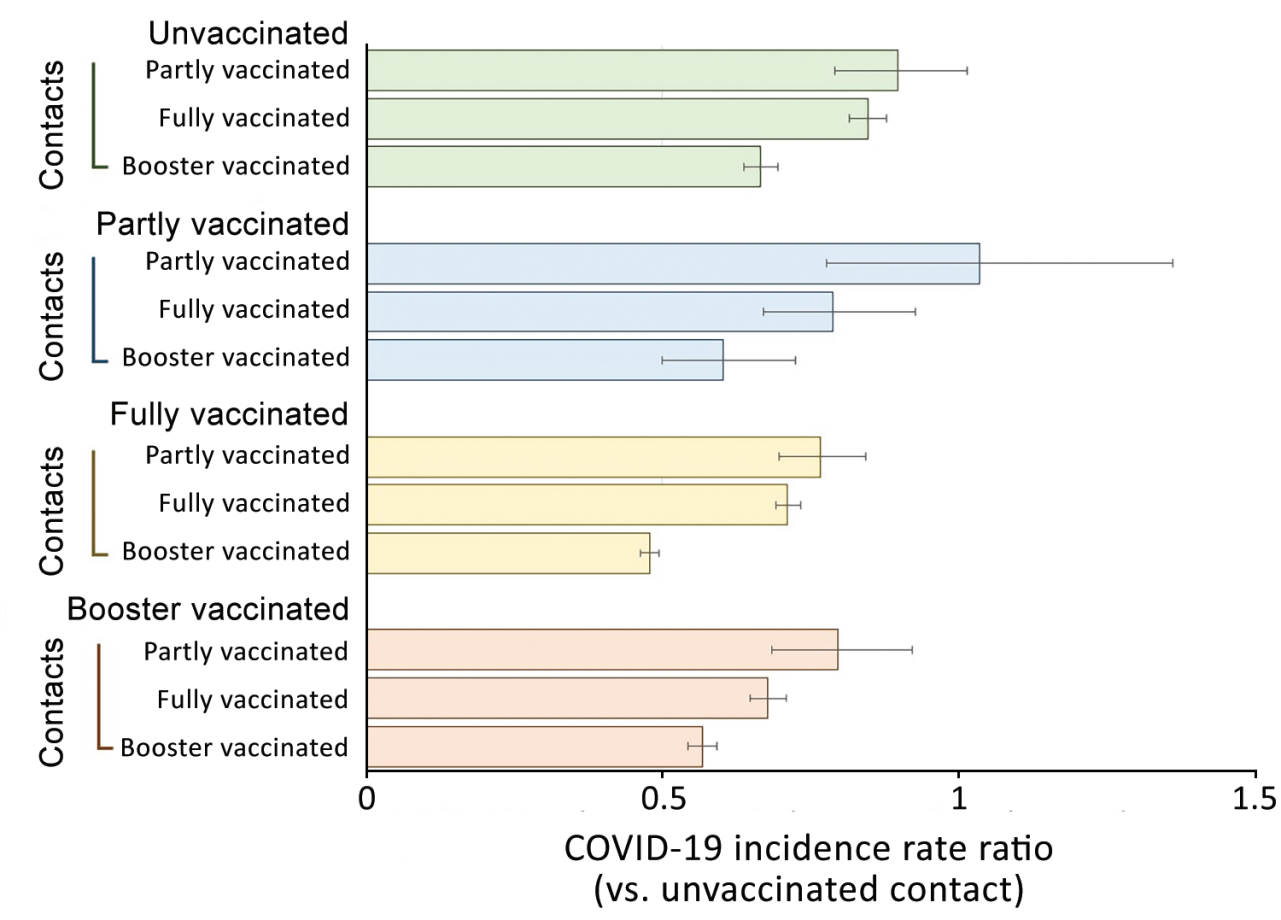
Vaccination status of household contacts relative to the vaccination status of SARS-CoV-2 Omicron variant index case-patients, South Korea, February 1–10, 2022. Header rows indicate vaccination status of index case-patients, and vaccination status categories for their contacts are displayed below. Error bars indicate 95% CIs.

Our findings offer evidence of improved protection against SARS-CoV-2 transmission when household contacts have received booster vaccinations. Transmission occurred in 36.7% (59,982/163,581) of the household contacts we studied, a percentage that falls within the range of results from similar studies in Denmark (29%–39%) and the United States (67.8%) (*4*,*5*). Another study demonstrated an association between booster vaccination with mRNA vaccines and protection against symptomatic Omicron infection (*6*). Consistent with these findings, our observations suggest that booster vaccination offers a higher level of protection against Omicron infection when household contacts are vaccinated and boosted.

The first limitation of our study is that surveillance did not clearly distinguish other potential sources of transmission within a household. Exposure outside the household might have led to some secondary cases. Second, difference in testing behavior based on vaccination status might have introduced bias into our findings. If unvaccinated persons have a different probability of getting tested compared with vaccinated persons, our results could be underestimating the true effectiveness of vaccines against household transmission; therefore, results of this study should be interpreted cautiously. Last, results based on such a large population might have produced statistical significance despite small effect size.

In summary, we provide real-world evidence to better understand the effect of booster vaccination in preventing household transmission of the Omicron variant of SARS-CoV-2. Additional studies are needed to determine the effectiveness of booster vaccination in regard to severe infections and deaths across different age groups. However, the higher SAR in younger household contacts we studied supports the need for public health initiatives to extend booster vaccination in younger age groups. 
